# Medicinal Cannabis and Implications for Workplace Health and Safety: Scoping Review of Systematic Reviews

**DOI:** 10.1177/21650799231157086

**Published:** 2023-04-19

**Authors:** Veronica O’Neill, Nektarios Karanikas, Adem Sav, Patricia Murphy

**Affiliations:** 1 Queensland University of Technology; 2 Independent researcher

**Keywords:** medical cannabis, adverse events, occupational health and safety, safety management

## Abstract

**Purpose:**

Although medicinal cannabis is prescribed for conditions such as pain, epilepsy, nausea and vomiting during cancer treatment, evidence about associated adverse side effects is still evolving. Because adverse events (AEs) might impact the performance of workers, it is important to consider their implications on workplace health and safety (WHS). This study aimed to map the types and prevalence of the AEs associated with medical cannabis and articulate how those events could impact WHS.

**Methods:**

A scoping review of systematic reviews and/or meta-analyses published between 2015 and March 2021 was performed to identify the AEs of medicinal cannabis in adults. Publications in English and full text available online were collected from Embase, MEDLINE, PsychINFO, PubMed, Scopus, and Web of Science.

**Results:**

Of 1,326 papers identified from the initial search, 31 met the inclusion criteria and were analyzed. The studies reported various AEs with the most predominant being sedation, nausea/vomiting, dizziness, and euphoria. Acute and chronic pain was the most prevalent disorder under review.

**Conclusions:**

Adverse events associated with the use of medicinal cannabis could increase workplace risks, including decreased alertness and reaction times, increased absenteeism, reduced ability to safely drive or operate machinery and an increased probability of falling. Focused research into the risk to workers and workplaces from the use of medical cannabis and related human performance impairment is urgently warranted.

## Background

The last three decades have witnessed scientific advancements toward using cannabis as a prescribed medical therapy (Dryburgh & Martin, 2020). Studies have encouraged pharmaceutical applications, while restrictions on the use, production, and distribution of cannabis endure due to its listing as a narcotic drug (Aguilar et al., 2018; Bridgeman & Abazia, 2017). However, treatment with medicinal cannabis is also associated with adverse events* (AEs), including nausea, vomiting, and dry mouth to more severe outcomes, including paranoia, tachycardia, and death (Abouk & Adams, 2018; Abuhasira et al., 2018; Andrade, 2016). In addition, transient psychosis and dependence has also been declared a high risk due an increased tolerance to the effects of cannabis (Arnold, 2021), often leading to behavioral and mood symptoms of withdrawal (Bonnet & Preuss, 2017). Adverse events can differ for each patient due to their age, underlying health conditions, gender, weight, patient compliance, interaction with other medications, food or vitamins, and overall health (U.S. Food and Drug Administration [FDA], 2018).

Cannabis use can lead to clinical impairment of psychological, cognitive, and physiological functioning (American Psychiatric Association, 2013). Research has demonstrated the significant impact of tetrahydrocannabinol (THC) cannabis on reaction time, motor co-ordination, ability to judge (Arkell et al., 2019; Hartman & Huestis, 2013; Lenné et al., 2010), and associations with road accidents (Brady & Li, 2014). A study indicated that there were 55% more industrial accidents, 75% higher absenteeism, and 85% more injuries for employees who tested positive for cannabis at work (National Institute on Drug Abuse, 2020). However, these studies mainly focused on cannabis for recreational use.

Despite acknowledgment of the potential adverse effects of cannabis, research into the potential AEs has not yet matured (Arnold, 2021), and there seems to be a dearth of long-term high-quality studies to confidently clarify patient safety aspects (MacCallum et al., 2018). Moreover, there is no globally accepted definition of impairment associated with medicinal cannabis and no agreement on how to measure its occurrence (Eadie et al., 2021). Yet, being cognizant of the adverse effects of medical cannabis and the probable impacts on human functioning is critical for workplaces. Under workplace health and safety (WHS) legislation in most countries, employers must provide a safe working environment while workers must also declare anything that could decrease their ability to work safely undertake work in a safe manner or threaten the health and safety of themselves and others affected by work activities (Government of Canada, 2021). Hence, there is an urgent need for occupational health (OH) providers and, in general, OH nurses, to support workplaces in making sure workers are fit for duty, and both workers and employers meet their legal obligations to avoid causing harm to others. This is particularly relevant for those taking medication, such as medicinal cannabis, due to adverse side effects and the subsequent risk of impairment.

As a first step toward addressing this gap, the current scoping review of systematic reviews and meta-analyses maps the types and prevalence of the AEs associated with medical cannabis and articulates how those events could impact WHS. This research contributes to the knowledge of medicinal cannabis by providing quality synthesis of current evidence to inform WHS policy and practice. This study could influence the review of existing policies and WHS legislation, including OH staff and employer responsibilities, return-to-work and rehabilitation programs, and worker compensation schemes.

## Methodology

To synthesize emerging evidence related to the AEs of medicinal cannabis, a scoping review methodology was chosen (Sucharew & Macaluso, 2019). To ensure transparency, we examined existing literature on the AEs of medicinal cannabis in humans was collected by using the guidelines of the Preferred Reporting Items for Systematic Reviews and Meta-Analyses (PRISMA; Liberati et al., 2009).

Time efficiency was gained through the identification of relevant systematic reviews and meta-analyses. These studies provided evidence of best quality as they employ strict scientific designs based on specific and reproducible techniques (Grant & Booth, 2009). The literature search was conducted for these types of studies across Embase, Medline, PsychINFO, PubMed, Scopus, and Web of Science (Table 1). The search strategy was completed under the guidance of a medical librarian and was peer-reviewed by all authors of this article.

**Table 1.** Medical Subject Headings for Literature Search

MeSH in abstract and/or title
(“Medic* marijuana” OR “medic* cannabi*” OR cannabinoid* OR “medic* THC” OR “medic* CBD” OR “medic* tetrahydrocannabinol”) AND (impair* OR react* OR activ* OR advers* OR effect* OR influen* OR impact* OR perform* OR function* OR mobil* OR behav*)
In title
*AND ((systematic AND review) OR “meta analysis”)*

As the use of medicinal cannabis is an emerging topic and requires timely access to evidence, the literature search was limited to the period between 2015 and March 2021. Articles were excluded if they were not systematic reviews and/or meta-analyses, were not available in English and online, did not research cannabis for medicinal purposes, did not clearly articulate AEs from medicinal cannabis, and did not include adults as research cohorts.

All studies (*n* = 1,326) were entered into EndNote 20 (Clarivate, 2021) and duplicates were removed. The abstracts and titles of the 481 remaining entries were initially screened by the first author (VO) and classified into the groups of *Excluded, Included*, and *Maybe*. The first author randomly selected 30 of the screened articles from within those three groups and split them into three sets for moderation by the rest of the authors (PM, AS, and NK). Each author reviewed 10 articles, and any discrepancies were resolved through discussion and consultation. After establishing confidence in the reliability of screening, VO and PM independently screened all 481 abstracts. Discrepancies from this screening were resolved through consensus between the two researchers, leading to the exclusion of 364 articles and inclusion of 117 publications for eligibility assessment.

**Figure 1.**
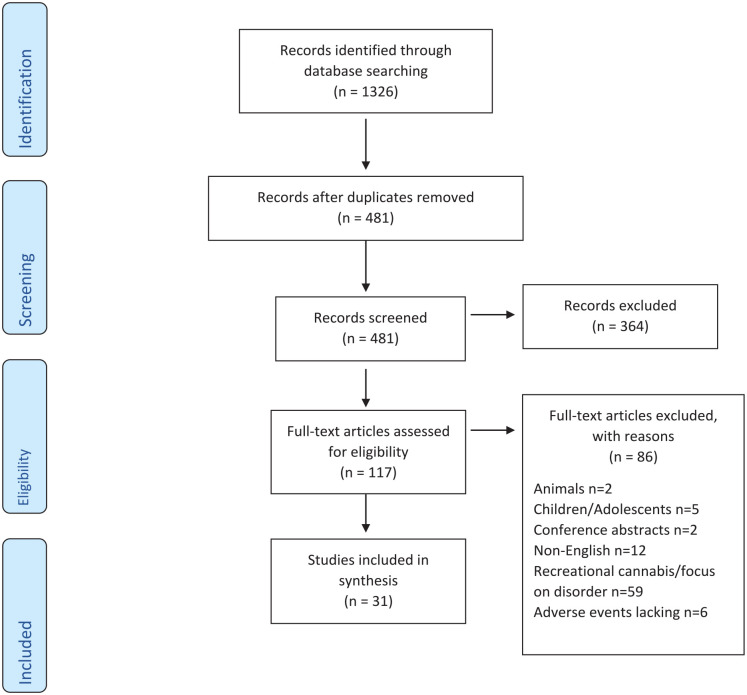
PRISMA Flow Diagram

After retrieving the full texts of the 117 articles, an additional moderation was undertaken by randomly assigning 10 papers to PM, AS, and NK for eligibility. Following the resolution of discrepancies through consultation and the achievement of consensus, VO assessed all 117 articles. This step resulted in the exclusion of 86 articles. As this is a scoping review, no formal quality appraisal was required to evaluate systemic errors within the methodologies employed, precision of results and external validity (Grant & Booth, 2009). Figure 1 presents the literature screening flow.

The data extracted from each systematic review and meta-analysis study included information about the year and country of publication, aims of the review, specific populations examined, number of original papers analyzed, and key findings associated with AEs of medicinal cannabis. All AEs were provided with a code, where *AExxx* and *SAExxx* represented AEs and SAEs (SAEs), respectively, with *xxx* their unique identification number. During the review of the studies, the research team noticed it was unclear what criteria the authors of each paper followed to classify an adverse event as serious. Excluding easily recognizable serious events like *death*, a *life-threatening medical event*, and *suicidal behaviors*, the classification of events as serious or not was not consistent in the data. For example, *disorientation* and *urinary tract infection* were classified as serious in one study (Mohiuddin et al., 2020) and not serious in other articles (Allan et al., 2018; Wong et al., 2020).

Next, as the terms used to describe AEs varied across studies, an additional review of all events was undertaken by VO and PM. During this process, all similar terms were grouped together. Any discrepancies were resolved through consultation and consensus. Moreover, the lowest and highest percentages of participants who experienced AEs were recorded. Finally, to yield an overall picture per article, we calculated the average percentages of the participants who experienced the AEs, identified within each publication.

## Results

### Study Characteristics

The principal aim of all 31 studies was to review the efficacy and safety of medicinal cannabis across a range of disorders. These disorders were investigated across 648 articles contained in the systematic reviews/meta-analyses analyzed. Of the 31 studies, 16 reviewed patients experiencing acute, chronic, and non-cancer pain; five reviewed the symptoms of cancer treatment and pain; four studies examined psychiatric disorders; and six reviewed symptoms associated with Crohn’s disease, multiple sclerosis (MS), fibromyalgia, and spinal cord injury (SCI).

Although, most of the studies (*n* = 26) focused on one primary condition, two studies reported on three areas of interest (i.e., nausea/vomiting, chronic pain, and spasticity) (Allan et al., 2018; Whiting et al., 2015), one focused on neurological, psychiatric, and healthy adults in their review (Dos Santos et al., 2020), and one reviewed a range of conditions for patients older than 50 years (Velayudhan et al., 2021). In addition, only one study (Hindley et al., 2020), focused on healthy volunteers who were subjected to medicinal cannabis in anticipation of AEs.

The studies were not examined to investigate occupational settings nor the potential interactions and effects of additional prescribed medication on medicinal cannabis.

### Cannabis Type and Administration

All studies utilized cannabis-based medication to gauge the efficacy on the symptoms under treatment and/or the safety of medicinal cannabinoids, including synthetic cannabinoids (Nabilone and Levonantradol), Nabiximol, a plant-based THC:CBD spray (Sativex) and the man-made Dronabinol. Regarding administration, 35% of the studies focused on one route, such as capsules (Abo Youssef et al., 2017; Tateo, 2017; Velayudhan et al., 2021; Wang et al., 2019; Wong et al., 2020), smoking/inhalation (Deshpande et al., 2015; Mücke et al., 2018), and oral-mucosal (Allan et al., 2018; Boland et al., 2020; Darkovska-Serafimovska et al., 2018). The rest included a range of administration routes, such as capsules and intramuscular injections (Gazendam et al., 2020; Stevens & Higgins, 2017), capsules and oral-mucosal (Aviram & Samuelly-Leichtag, 2017; Dos Santos et al., 2020; Fitzcharles et al., 2016), and capsules and smoking/inhalation (Ghabrash et al., 2020; Kafil et al., 2018; Kurlyandchik et al., 2020; Schussel et al., 2018; Wilkinson et al., 2016).

Only one study reviewed smoking/inhalation and oral-mucosal (Rabgay et al., 2020), one study reviewed capsules, inhalation/smoking, and intramuscular injection (Hindley et al., 2020), and a further study reviewed capsules, inhalation/smoking, oral-mucosal, and intramuscular injection (Whiting et al., 2015). The final six studies reviewed capsules, inhalation/smoking, and oral-mucosal as routes of administration (Johal et al., 2020; Longo et al., 2021; Lynch & Ware, 2015; Mohiuddin et al., 2020; Nabata et al., 2020; Nugent et al., 2017). Cannabis doses were not comparable across any of the studies.

### Adverse Events

All 31 studies identified and clearly reported on individual AEs (*n* = 133), with 12 studies additionally reporting SAEs (*n* = 20). Twenty-two studies identified *nausea* as a consistent AE reported by 67% of the subjects and *dizziness* was listed across 20 studies with 67% of the subjects. The third most frequently reported AE across 16 studies was a *dry mouth*, while *vomiting* and *fatigue* were reported across 14 studies each. Although the AEs above were cited across a range of studies, 71 AEs (53%) were listed only within a single study. The percentage of the studies which each of the 31 systematic reviews/meta-analyses found reporting AEs ranged from 4% (Velayudhan et al., 2021) to 100% for two systematic reviews that purported AEs across all studies (Schussel et al., 2018; Wang et al., 2019).

**Figure 2.**
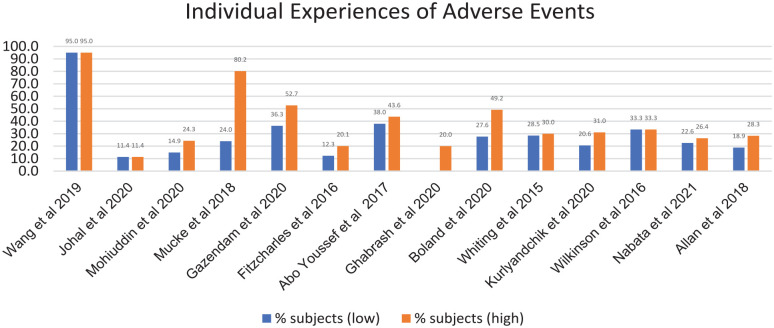
Range of Adverse Events Experienced by Subjects

Only 45% of the 31 studies (*n* = 14) recorded for all original research reviewed the proportion of participants experiencing AEs (Figure 2) with percentages ranging from 11.4% of subjects with chronic non-cancer pain (Johal et al., 2020) to 95% of subjects with cancer cachexia (Wang et al., 2019).

In total, 12 studies identified SAEs, all of them regarding participants experiencing pain. The most frequently reported SAEs were *urinary tract infection, pneumonia, fracture from fall due to dizziness*, and *suicidal ideations.* Sixteen cases of SAEs (*n* = 80%) were reported within one study only, including *trans-ischemic attack, death*, and *seizure* (Mohiuddin et al., 2020), *suicidal behaviors* (Nugent et al., 2017), and *serious disorientation, intolerable confusion*, and *severe euphoria* (Wong et al., 2020).

**Figure 3:**
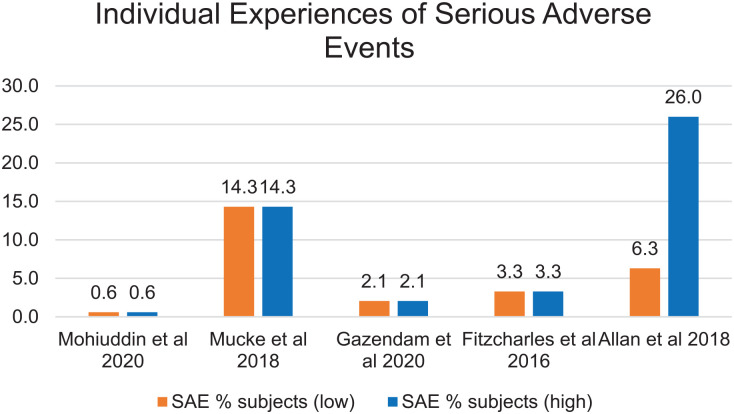
Range of Serious Adverse Events Experienced by Subjects *Note.* SAE = serious adverse events.

The greatest incidence of SAEs (i.e., 15 of 16 articles) was found in a study on chronic neuropathic pain (Mücke et al., 2018) and 83% of the studies on post-surgical subjects reported SAEs (Gazendam et al., 2020). The least reported incidents of SAEs were in the study that focused on subjects with chronic pain (Nugent et al., 2017). Of the 12 systematic reviews that identified SAEs, only five noted percentages of individuals affected (Figure 3).

After grouping the AEs reported with different names, sedation presented the highest incidence, with 53 referenced times across the original articles (Table 2). Experiences of *nausea* (*n* = 37), *dizziness* (*n* = 33), and *euphoria* (*n* = 26) were followed by *gastrointestinal* (*n* = 22). The events reported only once were death, rash, sweating, social stigma, and cancer. Other AEs included cognitive impacts, such as *confusion* (*n* = 18), *impaired concentration* (*n* = 16), and *impaired memory* (*n* = 11). Physiology-related concerns included *ataxia* (*n* = 11), *asthenia* (*n* = 11) and *muscular/skeletal impact* (*n* = 10). Psychological-related AEs included mood/anxiety (*n* = 16), such as *mania, depression, dysphoria*, and *suicidal behaviors* (*n* = 3).

**Table 2.** Incidence of Combined Adverse Events

Combined adverse events	Incidence of event occurring across studies
Sedation	53
Nausea/vomiting	37
Dizziness	33
Euphoria	26
Gastrointestinal	22
Dry mouth	18
Confusion	18
Impaired concentration	16
Cardiac (inc. increased/decreased heart rate [HR])	16
Psychological—mood/anxiety	16
Psychosis	14
Headache	12
Ataxia	11
Impaired memory	11
Asthenia	11
Muscular/skeletal impact	10
Respiratory	10
Nutritional disturbances	10
Not described	10
UTI	8
Eye irritation	8
Seizures	7
Agitation	7
Sleep problems	7
Pharyngolaryngeal discomfort	7
Dysphoria	7
Myalgia	5
Dysgeusia	4
Pruritis	3
Suicidal behaviors	3
Liver/gall bladder upsets	3
Pyrexia	2
Incapacitation	2
Dependence	2
Life-threatening medical event	2
Psychomotor	2
Tinnitus	2
Speech disorders	2
MS relapse	1
Rash	1
Sweating	1
Cancer	1
CNS disturbances	1
Viral infection	1
Social stigma	1
Burning sensation	1
Death	1

*Note.* MS = multiple sclerosis; CNS = central nervous system; UTI = urinary tract infection.

## Discussion

A range of AEs is attributed to the use of medicinal cannabis, which relates to implications of the physical, cognitive, and physiological states of individuals. Interestingly, there are significant differences in the percentages of affected individuals reported per original research that was considered in the systematic reviews and meta-analyses included in this study. This variation can be explained through pharmacodynamics, such as variation in receptor numbers and structure in an individual, and pharmacokinetics, including the differences in drug concentration on absorption, metabolism, and excretion, whereby different amounts of drugs reach the body’s receptor sites.

For example, body size is a key influence on the response of drugs in the system (Higgs et al., 2019). This is due to bigger frames having a larger vascular system; therefore, the same concentration of a drug will be diluted in a larger blood volume. Indeed, fat-soluble drugs, such as THC, would be absorbed into the body fat, and their period of action would be longer for a person who is obese as opposed to a person who is lean (Le, 2020). Age is a further pharmacokinetic factor (Higgs et al., 2019). Reasoning for this factor includes body composition whereby age increases the relative amount of fat to lean tissue (Ruscin & Linnebur, 2021). In addition, kidneys are less effective at excreting drugs into urine, and the liver becomes smaller and less efficacious.

In general, AEs can differ for each patient due to their age, underlying health conditions, gender, weight, patient compliance, interaction with other medication, food or vitamins, and overall health (FDA, 2018). Therefore, while there is a considerable range of AEs that can arise from taking medicinal cannabis, how to manage and accommodate these safely, especially during work activities, can be a challenging proposition for both the employer and the worker.

## Implications for the Workplace

Apart from the obligations of employers to provide a healthy and safe working environment, workers are also required to be fit for their role to minimize any risk to self or others and to perform their duties safely (Commonwealth of Australia, 2021). The findings from this scoping review show that workers who have been prescribed medicinal cannabis could experience a broad spectrum of AEs, which in turn can cause impairment in their functioning. Depending on the type of cannabinoid, the route of administration and the tolerances of the individual, such effects have the potential to jeopardize WHS.

Due to the lack of consistency in terminology and classification of (serious) AEs, the following sections focus on the most frequent of the AEs listed in Table 2, which have been categorized for the purposes of this study. Although the grouping of events, irrespective of reported severity level, serves the scope of this study, they should still be viewed with caution due to the inconsistencies and variability the authors observed in the way those events and their prevalence were reported across the publications analyzed. Furthermore, the possible effects of the less frequent events listed in Table 2 should not be underestimated as their incidence can vary across individuals, as discussed above. Notably, the coexistence of more than one of those AEs in individuals and their interactions can increase the cumulative risks of negative consequences within workplaces and beyond.

### Sedation

In this study, *sedation* was the leading adverse event identified (*n* = 53). Similar terms used across the studies were *fatigue, somnolence, tiredness, drowsiness, sleepiness*, and *lethargy*. Indeed, *sedation* can influence the ability to remain alert and focused, lead to reduced reaction times when responding to a situation, and impact the decision-making capacity (Phillips et al., 2015). Consequently, *sedation* at work can have ramifications for both the individual and the workplace as it can impact a worker’s physical and mental health. For example, poor work-related performance can increase the risks of task execution errors and incidents, which in turn, can increase the likelihood of injury to self and others, rate of mortality, and inflated costs for managing lost time (Sadeghniiat-Haghighi & Yazdi, 2015; Workplace Health and Safety Queensland, 2020).

### Nausea/Vomiting

*Nausea/vomiting* was the second most frequently reported impact (*n* = 37). While there is a difference between the sensation of *nausea* being discomfort and the urge to vomit, and *vomiting* being the final forced action, they are often described together and can range from mild to severe (U.S. National Library of Medicine, 2021). Although mild symptoms can be unpleasant they can be easily managed. Yet, when symptoms become severe, the quality of life can be negatively affected (Hasler & Chey, 2003). Indeed, it has been noted (Hasler & Chey, 2003), that *nausea* and *vomiting*, in an acute setting, are a means to protect a person following the ingestion of harmful substances. When symptoms are severe, they can impair a person’s ability to function and cause fatigue, weight loss and dehydration.

Furthermore, nausea/vomiting can lead to dehydration which is associated with weakness, fatigue, dizziness, headache, and thirst (European Hydration Institute, 2013). Those symptoms can cause impairments within the workplace. For example, it has been reported that dehydration in the workplace resulted in impaired decision-making and cognitive capacity and created risk of poorer work performance and increased preventable errors (Lamaire et al., 2010). In addition, weakness and dizziness can increase the risk of injury to worker due to an increased likelihood of a fall, possibly resulting in bone fractures (Velayudhan et al., 2021). Moreover, dehydration can also cause headaches that impact work efficiency, reduce productivity, and require short-term absence from the workplace (Simic et al., 2020).

### Dizziness

The third highest incidence in Table 2 was *dizziness* (*n* = 33), which was reported separately, although it can comprise one of the symptoms of *nausea/vomiting*. Similar terms identified across the studies included *light-headedness, feeling faint, vertigo*, and *dizziness/light-headedness.* The term *dizziness* is used to describe either a sensation in the head causing light-headedness, presyncope, vestibular disturbance, or disequilibrium causing a disturbance in co-ordination or balance (Post & Dickerson, 2010). However, the descriptions included in the studies reviewed suggest dizziness was associated more with the sensation of light-headedness rather than the other examples (Post & Dickerson, 2010).

Although we could not identify dizziness-related publications specifically for WHS, referred effects of this adverse event on the employment and daily living activities has previously been identified (San Filippo, 2017). These included an increased risk of injury due to a fall, inability to drive due to safety concerns and reduced capacity to concentrate and process tasks (San Filippo, 2017). Such effects can impact productivity levels, inflict additional costs for businesses and threaten workplace safety. Also, significant occupational consequences by way of absenteeism due to dizziness have been reported (van der Zaag-Loonen & van Leeuwen, 2014).

### Euphoria

The fourth most frequently reported adverse effect was *euphoria*. This is the sensation frequently associated with smoking marijuana cigarettes for recreational purposes (Alcohol and Drug Foundation, 2020). It is therefore not surprising that the incidence of *euphoria* ranked highly in the list of effects (*n* = 26). Participants also described this sensation as *feeling high* and *feeling drunk*. It was noted that cannabis induces emotional cognitive distortion which amplifies sensory signals received by the brain (Leonard & Pathak, 2020), which can explain the feelings of being high, drunk, or euphoric. Decreased reaction times (Phillips et al., 2015) and an inability to operate machinery safely (Arkell et al., 2019; Canadian Centre for Occupational Health and Safety, 2018) have been identified as significant impacts of *euphoria*.

Especially regarding the *feeling drunk* adverse event, Villines (2019) provided an overview of the effects of alcohol and level of “drunkenness” and further described feelings of euphoria at blood alcohol content (BAC) 0.07 to 0.09, in which people are more talkative, less inhibited and euphoric. At BAC 0.09 to 0.15, people still describe being euphoric and reaction times are slower. As in several countries, the BAC limit for safe driving is 0.05 (i.e., 0.05 g of alcohol for every 100 ml of blood), the associations above regarding the description of similar sensations somewhat confirm the work suggesting that *euphoria* in the workplace can be unsafe (Arkell et al., 2019; Canadian Centre for Occupational Health and Safety, 2018; Phillips et al., 2015).

## Limitations

The findings of this research are constrained by several limitations. As a scoping review, this study did not include a quality assessment of the publications analyzed. This may have decreased the objectivity of the research as the studies selected were based on availability and criteria related to the scope of this research rather than quality deductions to generate conclusions (Grant & Booth, 2009). Another limitation is the lack of investigation and synthesis of dosage, type of cannabinoid and route of administration. This was beyond the scope of this study and many of the papers included did not specifically mention synthesis of dosage, type of cannabinoid and route of administration. This would have been advantageous to derive the magnitude and duration of each adverse event and generate more tangible results for implications on WHS. In addition, it is noted that the synergistic effects of drug-to-drug interactions, especially between cannabidiol and conventional pharmacotherapies, can amplify adverse effects (Arnold, 2021). A final limitation relates to the subjective responses of the participants of the original research and the lack of clinical data. Although the latter cannot be directly obtained for experienced symptoms and sensations, we cannot ensure that the reported adverse event data were consistent, reliable, and always quantifiable (Lang & Wilkerson, 2008). Nevertheless, although we believe the limitations stated above did not threaten the objectives of this study, they reflect research gaps and could constitute opportunities to standardize future studies on the AEs of medicinal cannabis.

## Conclusion

Risks associated with the adverse effects in the context of WHS discussed in this study include, lower levels of alertness, reduced reaction times, increased absenteeism, decreased ability to safely drive or operate machinery and an increased probability of falling. Therefore, the AEs and their ramifications must be considered and proactively addressed to ensure a healthy and safe work environment. This will require a multidisciplinary approach with critical input from OH staff, including OH nurses, as they are in an ideal role to work alongside the workplace in assessing the risk of impairment connected to safety-sensitive roles (Phillips et al., 2015). However, well-defined policies will need to be established and implemented to guide best practice as this contentious topic continues to evolve.

With advancements in the use of medicinal cannabis for chronic health conditions and an aging global workforce with greater access to medicinal cannabis to manage pain and disability, a more comprehensive understanding of the implications of the effects of medical cannabis to WHS is critical. Further high-quality research is recommended with a focus on medicinal cannabis and AEs that impact WHS, including measurements of the intensity and duration of human functioning impairments associated with medicinal cannabis.

**Applying Research to Occupational Health Practice**

We identified a number of potential risks associated with the adverse events from the use of medicinal cannabis. From an occupational health and safety perspective, the adverse events and their ramifications must be detected and proactively addressed. Occupational health staff are in an ideal place to operate in concert with the workplace in assessing the risk of impairment connected, in particular, to safety-sensitive roles.
